# Indian Model of Integrated Healthcare (IMIH): a conceptual framework for a coordinated referral system in resource-constrained settings

**DOI:** 10.1186/s12913-023-10454-2

**Published:** 2024-01-09

**Authors:** Moonis Mirza, Madhur Verma, Arun Aggarwal, Sidhartha Satpathy, Soumya Swaroop Sahoo, Rakesh Kakkar

**Affiliations:** 1 Department of Hospital Administration, All India Institute of Medical Sciences, Bathinda, Punjab 151001 India; 2 Department of Community and Family Medicine, All India Institute of Medical Sciences, Bathinda, Punjab 151001 India; 3 Department of Community Medicine and School of Public Health, Post Graduate Institute of Medical Education and Research, Chandigarh, 160012 India; 4 Department of Hospital Administration, All India Institute of Medical Sciences, New Delhi, India

**Keywords:** Continuum of care, Integrated care, Universal Health coverage, Equity in Health, Time to care

## Abstract

**Introduction:**

With the escalating burden of chronic disease and multimorbidity in India, owing to its ageing population and overwhelming health needs, the Indian Health care delivery System (HDS) is under constant pressure due to rising public expectations and ambitious new health goals. The three tired HDS should work in coherence to ensure continuity of care, which needs a coordinated referral system. This calls for optimising health care through Integrated care (IC). The existing IC models have been primarily developed and adopted in High-Income Countries. The present study attempts to review the applicability of existing IC models and frame a customised model for resource-constrained settings.

**Methods:**

A two-stage methodology was used. Firstly, a narrative literature review was done to identify gaps in existing IC models, as per the World Health Organization framework approach. The literature search was done from electronic journal article databases, and relevant literature that reported conceptual and theoretical concepts of IC. Secondly, we conceptualised an IC concept according to India's existing HDS, validated by multiple rounds of brainstorming among co-authors. Further senior co-authors independently reviewed the conceptualised IC model as per national relevance.

**Results:**

Existing IC models were categorised as individual, group and disease-specific, and population-based models. The limitations of having prolonged delivery time, focusing only on chronic diseases and being economically expensive to implement, along with requirement of completely restructuring and reorganising the existing HDS makes the adoption of existing IC models not feasible for India. The Indian Model of Integrated Healthcare (IMIH) model proposes three levels of integration: Macro, Meso, and Micro levels, using the existing HDS. The core components include a Central Gateway Control Room, using existing digital platforms at macro levels, a bucket overflow model at the meso level, a Triple-layered Concentric Circle outpatient department (OPD) design, and a three-door OPD concept at the micro level.

**Conclusion:**

IMIH offers features that consider resource constraints and local context of LMICs while being economically viable. It envisages a step toward UHC by optimising existing resources and ensuring a continuum of care. However, health being a state subject, various socio-political and legal/administrative issues warrant further discussion before implementation.

## Background

Despite constant improvement in the Socio-demographic Index, there is still enough scope in the Indian healthcare system. This is because certain health indicators of India depict significant disparities from the economically advanced Organization for Economic Cooperation and Development (OECD) countries and are concerning. For instance, India's average life expectancy is around 70.8 years compared to 80.4 years in OECD countries. Indian maternal mortality ratio (103 per 100,000 live births) and infant mortality rate (25.5 per 1,000 live births) are way higher than that of the OECD (9.8 and 4.1) [1]. Further, approximately 122 Indians per 100,000 lose their lives due to poor quality of care and accessibility each year, and the estimates are poorer than many other countries [2, 3], The improvement in the quality of the healthcare delivery system (HDS) was envisaged through the Bhore Committee Report (1946) using the existing 3-tier system (Sub Centres (SC), and Primary Health Centres (PHCs) in villages, community blocks as Community Health Centres (CHCs), district hospitals, and tertiary care hospitals) [4]. Despite substantial progress, there were 157935 and 3894 SCs, 24935 and 6118 PHCs and 5480 and 584 CHCs functional in Indian rural and urban areas by March 2022 [5]. The HDS is further supported by a total of 134224, 184175, 109937, 162749 and 224679 hospital beds at the level of PHC, CHC, Sub District Hospitals, District Hospitals and Medical Collages [5]. However, the 2019–20 Rural Health Statistics depicts very low adherence to standards as mandated by the Indian Public Health Standards (IPHS) at all three levels of HDS throughout the country [6, 7]. This is attributed to the modest 2.1% of the Gross Domestic Product (GDP) allocated to the healthcare sector, leading to a lower density of medical practitioners (1.34 doctors per 1,000 population), lesser number of hospital beds (5.3 beds per 10,000 population) and higher Out-of-pocket healthcare expenditure (OOPE) (50.60% of health spendings) [1]. With the escalating burden of chronic disease and multimorbidity in India, owing to its ageing population and overwhelming health needs, the Indian HDS is under constant pressure due to rising public expectations and ambitious new health goals.

We constantly need to manage the patients requiring acute and immediate interventions appropriately to decrease the overall disease burden. Such management may not be possible at lower levels of HDS that call for referral to higher centres. However, the lack of accountability and monitoring mechanisms for the referral process has led to uncoordinated self-referrals, resulting in overused and overburdened tertiary care facilities (which could be managed in primary care), thus compromising the quality of care [8, 9]. The unwarranted fragmentation in HDS has also increased medical errors, misdiagnoses, and provider burnout, accounting for nearly 60% of the cross-regional variation in care, with substantial economic implications [10, 11]. Poor access to qualified care only represents the tip of the iceberg, and poor health promotion, patient education and prevention of chronic disease through timely lifestyle interventions are other deprivations attributed to inadequate quality of care that ultimately lead to increased disease burden and overwhelmed health systems. While health promotion, patient education and prevention of chronic disease through lifestyle interventions remain the mainstay of the primary healthcare system, tertiary care is rather about ‘disease control’. Therefore, the primary, secondary and tertiary care centres must work in coherence to ensure continuity of care, thus calling for optimising health care through Integrated care (IC).

The IC has been defined by the World Health Organization (WHO) as the *“management and delivery of health services such that people receive a continuum of health promotion, health protection, and disease prevention services, as well as diagnosis, treatment, long-term care, rehabilitation, and palliative care services through the different levels and sites of care within the health system and according to their needs.”* [12]. The IC enhances the patient experience and achieves greater efficiency and value in HDS [13]. For this, IC must demonstrate two fundamental characteristics. *First,* it must unite the fragmented HDS in its design and execution. *Second,* it should enhance the coordinated and continuous care for those in need [14]. This reiterates the concept of person-centred coordinated care that puts the control of decision-making in the hands of recipients to plan their care and works in bringing together services to achieve the outcomes crucial to them [15]. Previous studies analysed existing IC models and highlighted the need for change at the system and governance level. Developing and rapidly implementing IC models requires resources, expertise, and policy changes. The known barriers to implementing changed systems include a lack of clarification of roles and responsibilities, competition for funding, and challenges in engaging primary care providers [16]. Another critical challenge is to develop a sense of ownership and create an environment of collaboration between all the stakeholders [17]. The National Health Service (NHS) vanguard sites have been encouraged to develop new IC models after considering and adapting to the local and regional context [18]. The WHO’s global strategy for adopting people-centred IC services outlines a five-point plan emphasising health awareness, improving governance, reorienting the existing models, integrating the services, and providing an enabling environment for all stakeholders [19]. Based on these requirements, the WHO has proposed many critical forms of ICs, including horizontal integration, vertical integration, sectoral integration, people-centred integration, and whole-system integration [20]. However, there is evidence that the more severe the patient’s demand, the more suitable it may be to create a customised “fully integrated” model to manage their complex needs [21]. In the existing literature, IC models have been mainly developed and adopted in developed nations rather than low-middle-income countries (LMICs), including India [22]. Despite an organised 3-Tier HDS, India has not successfully developed or adopted any existing IC models. With this background, the present study attempts to review the applicability of existing models of IC and frame a customised IC model for developing countries like India that can fit and augment the existing healthcare delivery system.

## Methods

### Study setting

Provided the complexity of the domain, the framework was developed in two stages through a reiterative process of (1) a narrative literature review of the existing IC models classified by WHO and (2) group meetings to frame the customised IC model.

#### Literature search

The authors conducted a narrative literature review to identify globally existing conceptual and theoretical concepts regarding the individual, group, disease-based, and population-based IC models as classified by WHO and assess their fundamental principles, implementation barriers, and limitations of the models. The review was conducted in accordance with the current PRISMA guidelines [23]. The literature search was done from electronic journal article databases using search terms such as ‘integrated*,’ ‘integration,’ ‘levels of care,’ ‘health policy,’ and ‘integrated healthcare models*. We further explored reference lists of potentially relevant papers, books, and chapters written in English that reported conceptual and theoretical concepts related to IC. Initially, we did a title and abstract review, and those articles justifying our study objective were included. In cases where agreement could not be reached, a third reviewer was consulted, and we also emailed some prominent researchers in the field to seek further clarification. The full text of the articles was read, and relevant information (countries which adopted the IC models, the source of funding, salient features, and limitations) was compiled using Excel sheets.

### Data charting

#### Data collection

The study was framed in two steps. First, reviewing existing IC models as per their categorisation into the WHO framework into individual-based, disease-based, and population-based models. Additional information like country of origin, funding resources, salient features, and barriers/limitations were added to give a comparative framework so that the readers could understand the key points and limitations of all the IC models. Additional information was collected by snowball methods to maximise the information available on the subject.

#### Data analysis

The principal author (MM), a hospital administrator, conceptualised the first draft of the framework as a rudimental concept of integrated care according to the key features of the existing healthcare delivery system. To improve the content validity of the framework, we conducted multiple rounds of brainstorming and discussions with co-authors, and necessary changes were made to define the proposed patient pathway. Three senior co-authors, experts in the policy framing and healthcare delivery system, independently reviewed the paraphrasing and made suggestions as and were essential per national relevance. During multiple meetings between the experts, a discussion was held on synthesising the essential elements of a customised integrated care model. Based on these discussions, we refined the framework.

## Results

We initiated the process of model development following a narrative review. IC models were broadly categorised into Individual models of integrated care, Group and disease-specific models, and Population-based models (Table 1). Each model was reviewed in terms of country of origin, funding resource, salient features, and limitations were framed. Individual IC models mainly originated in the USA, England, and other countries under the OECD. The models were funded by government and non-government organisations. The individual IC models have been criticised for being time-consuming and costly [24–27]. Group and disease-specific models like the Chronic Care Model (CCM) and Program of Research to Integrate the Services for the Maintenance of Autonomy were adopted in developed countries like the USA, Sweden, Scotland, and Germany and were funded by the government. The limitation in the implementation of group and disease-specific models concerns the need to reorganise the entire healthcare system and prolonged delivery time focusing on specific age groups, thus affecting the program's application [28–30]. Population-based models like Kaiser Permanente are non-government and financers-based models which need to be enrolled by opting out of tax-based government coverage health system [31]. Based on the review, it was seen that the levels of HDS are planned per the complexity of the health conditions but have no physical or virtual integration to track the patients in terms of continuity of care that is sustainable at the same time and within the scope of existing infrastructure.

**Table 1:** Review of the existing models of Integrated care in the world

**Integrated care models**	**Country**	**Funding**	**Salient features**	**Limitations**
**Individual models of integrated care**				
American Case Management Association	USA	Both Government/Non-Government as Medicare and Medicaid	Promotes collaboration between patients, caregivers, nurses, social workers, doctors, and the community. Focuses on individualized communication to improve access to care through resource coordination [32]. The case manager assesses the patient's and caregiver's needs to establish a customized care plan, monitors the quality of care, and maintains communication with the patient and caregiver [33]. Evidence demonstrates that case management reduces hospital (re)admissions and enhances patient satisfaction.	The Cost-effectiveness of case management is still debated [24] and thus limits its acceptance in LMIC
Individual care plans	Countries under Organization for Economic Co-operation and Development (OECD)	Government As Medicare	Considered for patients with multimorbidity and long-term conditions. Care coordinators assess a patient's needs, design care plans, and organize multidisciplinary care delivery [25]. A personal budget (PB) is a type of intervention that emphasizes the patient's active involvement in their care. It comprises a sum of money allocated to the individual and used for various things in accordance with personal needs [34].	Individual care plans are time-consuming and costly to implement [24]. As a result, they necessitate precise eligibility requirements and rigorous beneficiary selection, thus limiting their acceptability to highly populated LMICs [25].
Patient-centred medical home(s)^2^(PCMH)	USA	Non-Government	PCMH provides an alternative to the primary care network by physician-directed groups with nurses as care coordinators assigned to particular medical homes. Patients act as partners in understanding the culture, unique needs, preferences, and values of patients with multi-morbidities and chronic diseases [35]. PCMH uses information technology and health information exchanges, as well as allocating interdisciplinary teams [24]. The PCMH has been able to cut hospital admissions by 20% and readmission rates by 12% among its beneficiaries [36].	PCMH has been chastised for being very fragmented, with delays in service delivery until reimbursement is rewarded [26].
Personal health budgets (PHB)	England	Government	PHBs focus on solving the ongoing needs of patients in terms of lived experiences by involving clinical practitioners' learned expertise to improve the quality of life [37]. A personal health budget is a monetary amount set aside by a person, or by their agent, and approved by the local integrated care system to meet that individual's needs for health and wellness.	PHB has been challenged for not being cost-effective in the long term for patients [38]. Further, Personal budgets have been accused to risk the fundamental principle of accessing healthcare based on clinical need and not the ability to pay [27].
**Group and disease-specific models **				
Chronic Care Model (CCM)	USA and Countries under OECD	Government and Non-Government	CCM focuses on integrated community-based longitudinal and preventive care, in place of acute and episodic care. CCM functions through productive interactions and establishing partnerships between a community-based proactive practice team and encouraging informed patients to participate in community programs. The success of CCM is projected mainly due to the bidirectional communications, multidisciplinary team approach, and encouraging self-care [39].	Barriers to the implementation of CCMs belong mainly to the patient’s will to change their behavior [28]. CCM is pitched to clinically oriented systems and is difficult to practice for the prevention and health promotion physicians. Also, slow response times from nurses and doctors, the need for regular training of staff [29], and patients may not actively contribute to self-care or may not have time for self-management support.
Program of Research to Integrate the Services for the Maintenance of Autonomy (PRISMA)	Canada	Government	PRISMA model was designed to integrate the health and social services needs of elderly and frail patients which later on became the part of a Quebec-wide program called *Réseau de Services Intégrés aux Personnes Âgées* (RSIPA) [30]. The model aims to serve as a single-entry point to the system and coordinate care for the elderly and frail population. The model maintains people's functional stability, lowers the severity of unmet demands, and lightened the load on caregivers. A joint health and social care governing board establishes the strategy and allots funds to the network. It has been observed that participants in the PRISMA program had lower readmission rates to hospitals [40].	Limitations with PRISMA, RSIPA, and similar programs are that the elderly population has to get enrolled in the program through case managers and meet the defined criteria for admission. [41] Also, for the implementation of PRISMA/RSIPA models, reorganization of the entire health care system is required which has affected the application of the program [30].
Chains of care model	Sweden	Government	The Sweden-based Chains [42] of care model was planned to connect screening components in a primary care facility, treatment plans developed in a specialty facility, and rehabilitation services offered in the community [43]. It acts by making use of contracts and aligns incentives to promote effective resource utilization.	Despite planned goals and activities, seven out of ten councils are unsure of the effectiveness of the development work. The most frequently cited causes of the failure include limiting vertical organizational structure and inadequate involvement of the local authorities [44].
Managed Clinical Network	Scotland	Government	**The managed clinical network** developed in Scotland moves from competition to cooperation [45] among the healthcare providers working in primary, secondary, and tertiary care, in a coordinated manner. Networks mainly work to improve service for patients with rare conditions or complex care needs. Clinical Networks are designed separately for a wide range of conditions ranging from Care of Burns in Scotland (COBIS), Children and Young People's Allergy Network (CYANS), Children with Exceptional Healthcare Needs (CEN), Cleft care Scotland, Network for Inherited Cardiac Conditions Scotland (NICCS), Inherited Metabolic Disorders Scotland (IMD), National Gender Identity Clinical Network Scotland (NGICNS), and so on [46]. Managed clinical networks offer better access to services with Improved coordination and Consistent advice for better care and prevention [45].	Improvement is not linear for all the conditions and age groups as the network mainly focused on adults, young people, and children [45]. Further progress was significantly slower than expected, which at times caused frustration due to a lack of knowledge about leading practice, as well as inexperience with change management.
Disease Management Programmes (DMPs)	Germany & Israel	Government	DMPs were introduced in the German health system to standardize nationwide programs regulating the entire duration of care in chronic conditions. While enrolment is voluntary, patients are required to adhere to the treatment goals and participate in self-management programs and disease-specific education [47].	Barriers in implement DMPs include a lack of budgetary allocations and prolonged delivery time compared to compensation [48].
**Population-based models**				
Kaiser Permanente	USA	Non- Government	With more than 9.6 million members across eight different states, Kaiser Permanente (KP) is one of the biggest health maintenance organizations in the USA. KP acts as an independent organization, separate from the government-provided healthcare delivery system. KP is a virtually integrated system comprised of three interconnected entities: a self-governing for-profit physician group (Permanente Medical Groups), a non-profit hospital system Kaiser Foundation Hospitals), and a non-profit health plan that covers insurance risks (Kaiser Foundation Health Plan). All three systems are mutually exclusive with regard to the purchasing and provision of services, but remain bound together by a single mission, combining systemic and normative integration [49].	Since patients have to choose either the tax-based government coverage or the KP system, benefits based on government universal coverage may not be provided to enrolled patients. Health coverage to enlisted individuals is based on health plans ranging from low coverage to high coverage, based on the co-payments [31]. Thus, the KP model will be difficult to adopt in resource-limited LMIC where financers and insurance-based coverage is limited
Veterans’ Health Administration	USA	Non-Government	Older adults with chronic diseases in the United States can receive integrated treatments from the Veterans Health Administration (VA). The VA owns and operates hospitals and employs clinicians to provide services within its network. The VA consists of 21 regionally based integrated service networks [50].	Only works within the network and thus cannot be implemented in the regions outside the VA integrated service networks
Integrated care in the Basque country	Basque	Government	In order to improve the outcome of care for chronic patients, Basque integrated care recognizes the interdependencies between primary care, social services, and hospitals to produce better results. Integrated care was provided using two different strategies. A bottom-up approach where primary and secondary care physicians were emphasized on coordination of care procedures. Integrated Healthcare Organizations (IHOs) were formed by combining hospital and primary care institutions. Important aspects of the model include simultaneous activation of all systems that aid integrated care. Units for Continuity of Care (CCU), established by IHOs to serve high-risk patients have enhanced coordination. CCUs are staffed with dedicated referral internists who are in charge of admitting and stabilizing chronic patients and transfer from the hospital to home, where they will subsequently be followed up on by their general practitioner. The use of strategies including patient education and information technology has been another factor in the success of the Basque integrated care strategy [51].	The primary care practitioners value the integrated system, but professionals at all the central levels impose barriers to implementation as lack of funding and political backing, time restrictions for consultations, and trouble juggling conflicting daily needs [52].

### Proposed solution

**Fig. 1:**
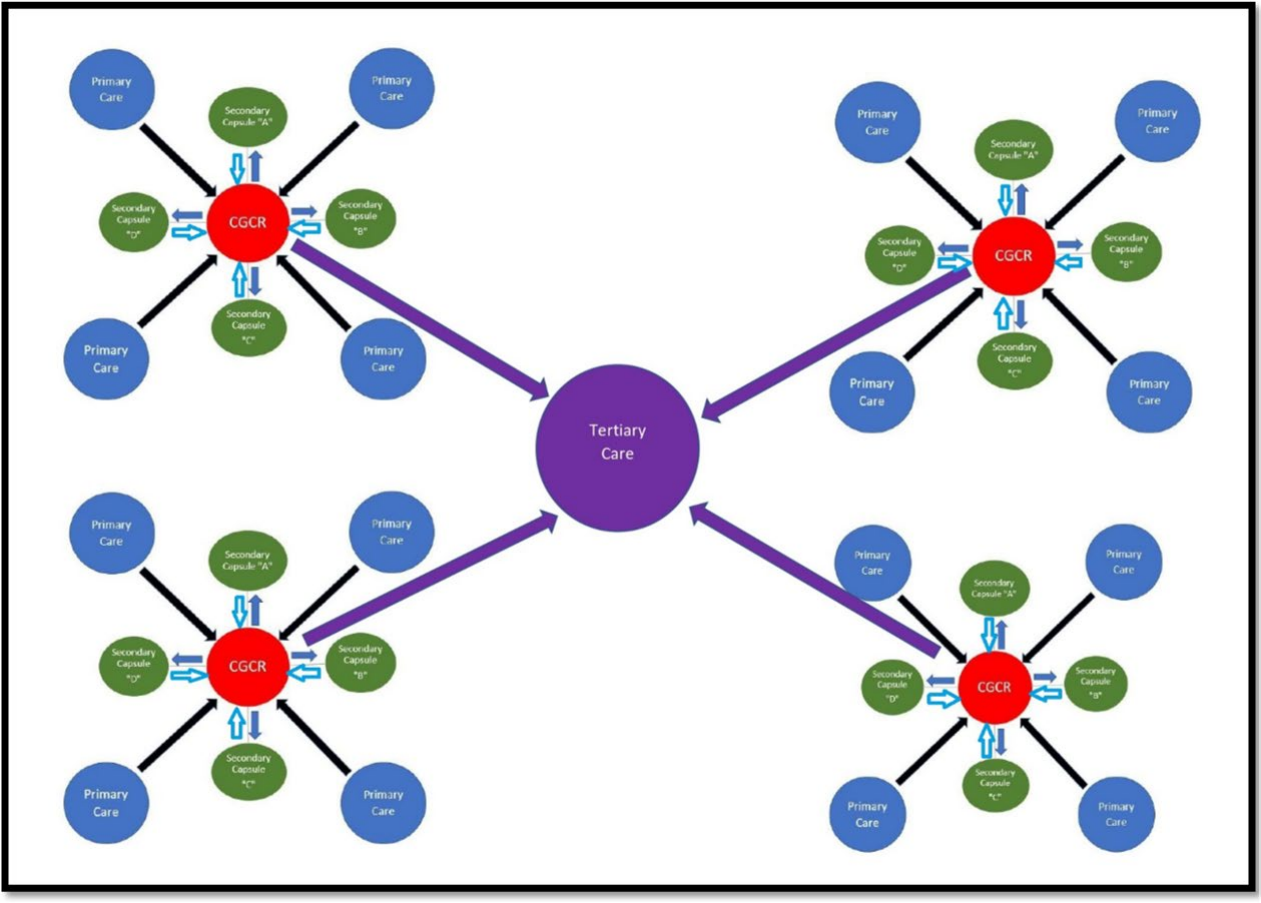
Proposed IMIH framework

**Fig. 2:**
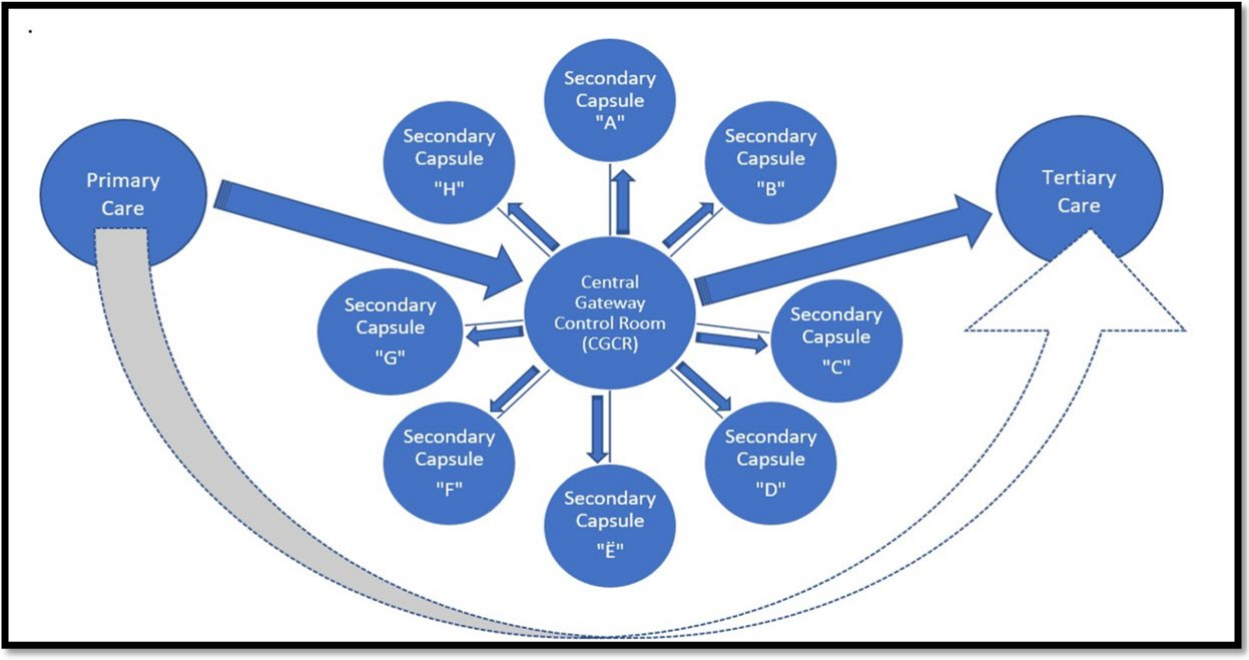
Functioning of Central Gateway Control Room in the IMIH

**Fig. 3:**
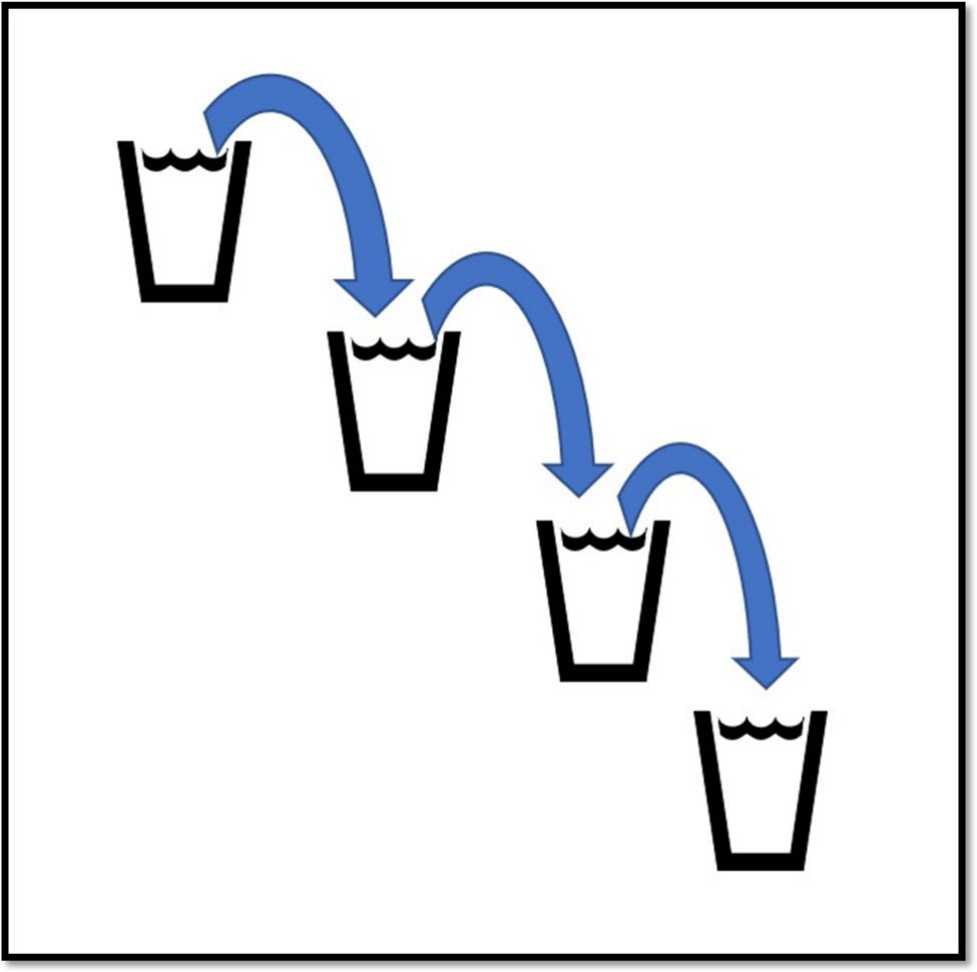
Bed allocation at secondary level capsules by bucket overflow method

**Fig. 4:**
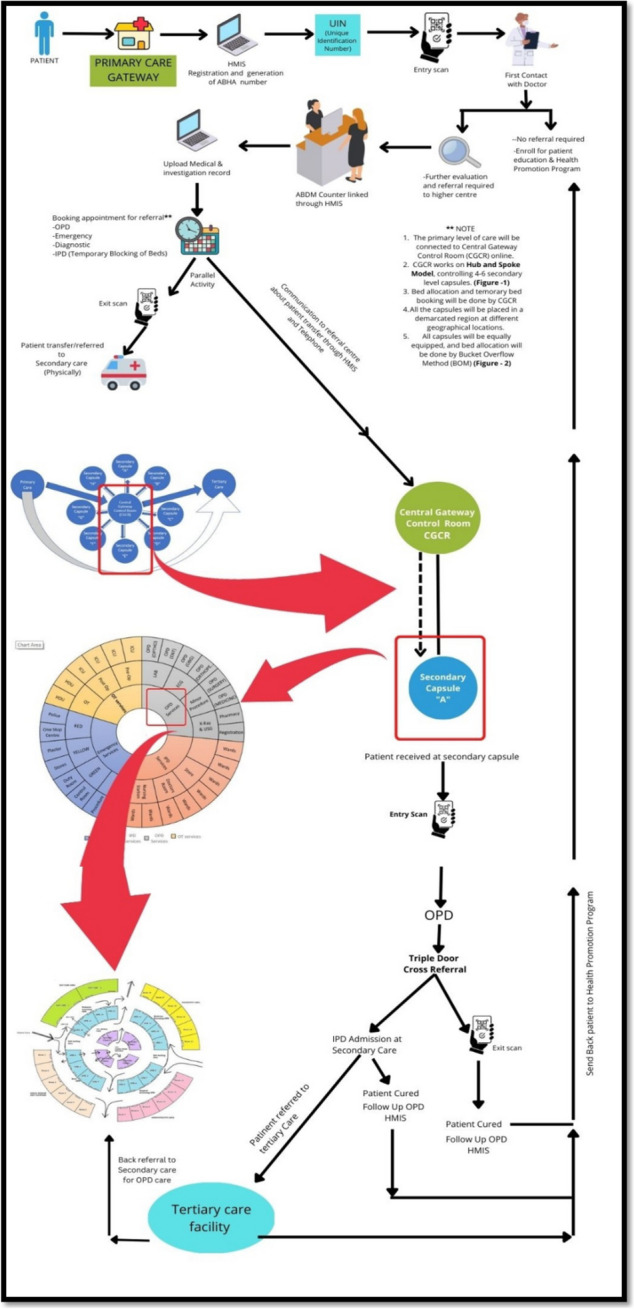
Patient pathway in the IMIH

**Fig. 5:**
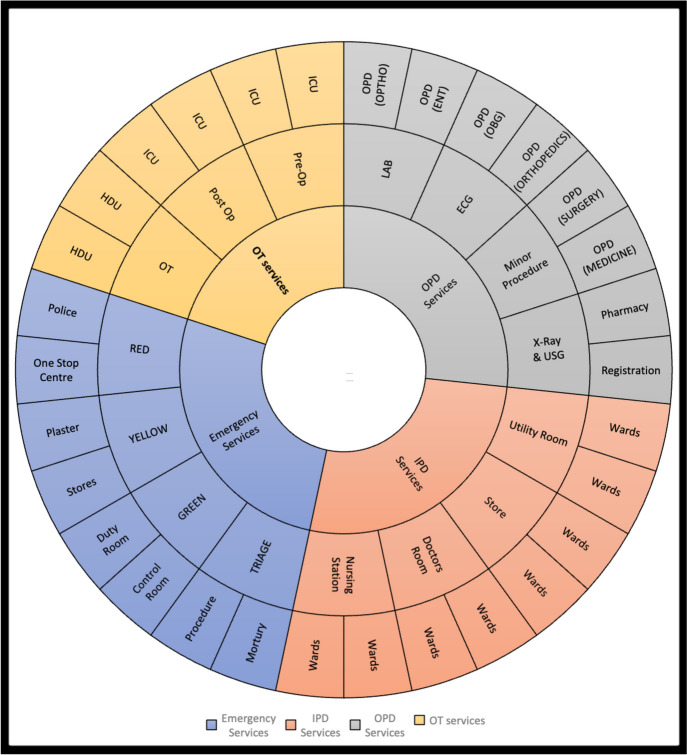
Layout of Secondary Capsules (SdC) in the IMIH

**Fig. 6:**
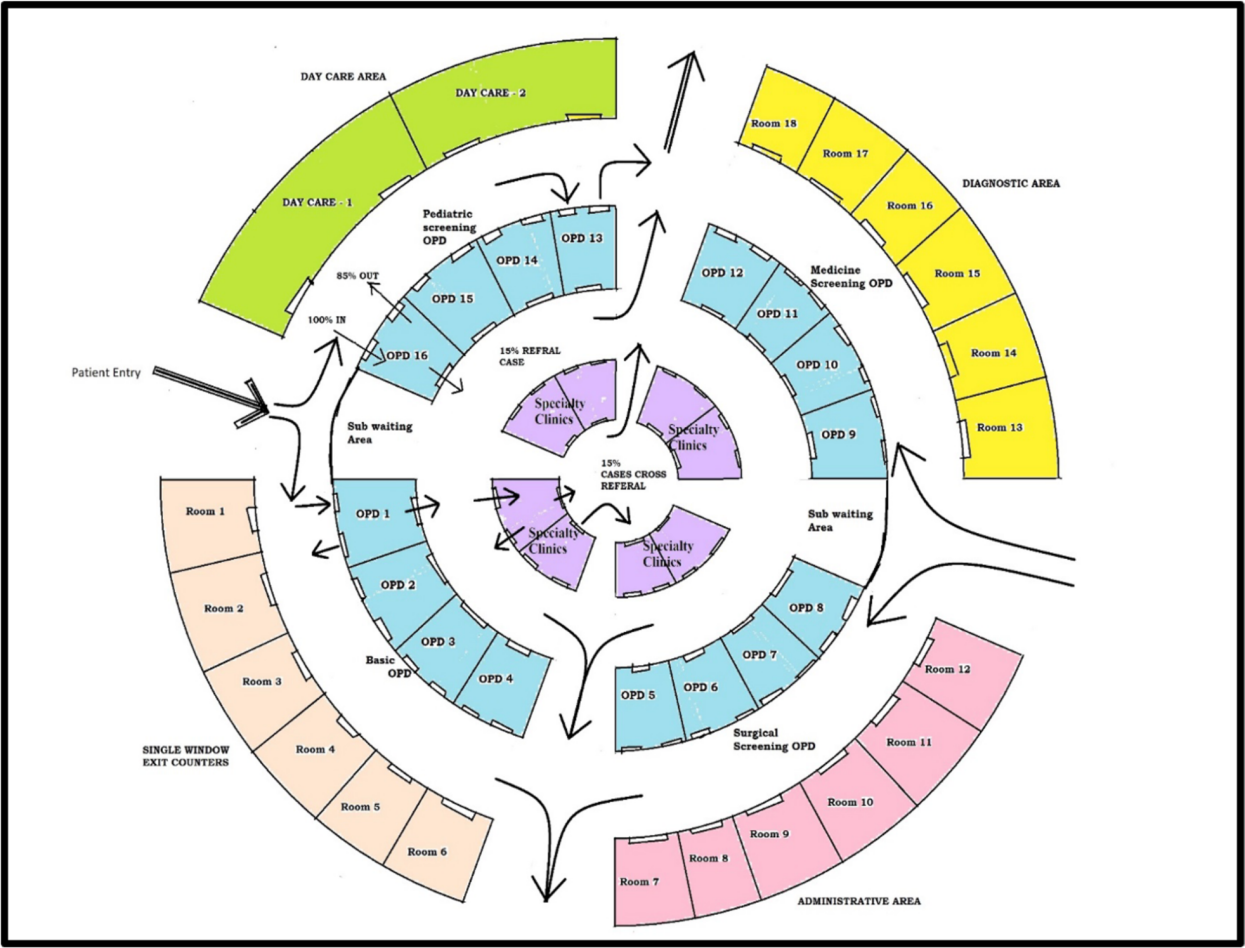
Triple-layered Concentric Circle OPD (TLCCO) design with Three Door OPD (TDO) in the IMIH

Considering the existing distribution of human resources, infrastructure, and planned patient load at each level of care, we propose a new model of Integrated care framework called the “Indian Model of Integrated Healthcare (IMIH).” The model has been planned at three levels of integration: Macro, Meso, and Micro levels Integration using the existing HDS as follows:

A Macro level of integration:
  The Macro Level of Integration will help in load-balancing by diverting patients in a coordinated manner through a gateway between 3 tiers of HDS using the Central Gateway Control Room (CGCR) within the states, which can be scaled up in later stages, to include the apex centres (Fig. 1). CGCR will be connected to the Secondary level grid with similar replicating capsules using the Hub and spoke model. Primary-level gateways will act as the first point of contact for initial evaluation and will divert the patients to CGCR in case of a requirement that calls for a referral.
  Existing Tools and schemes already rolled out by the Government of India like, Ayushman Bharat-Pradhan Mantri Jan Arogya Yojana, or PM-JAY (government-funded health insurance scheme), Ayushman Bharat Digital Mission (ABDM), Integrated Disease Surveillance Program (IDSP), Integrated Health Information System (IHIP), eHospital, e‐Shushrut, Electronic Vaccine, Intelligence Network (eVIN), Integrated Health, Information Platform (IHIP), National Health Portal (NHP), National Identification Number (NIN), Online Registration System (ORS), Mera Aspatal (Patient Feedback System), Health Management Information System (HMIS), will help in bridging the data gaps across the chain of treatment and will act as the backbone for developing Patient Surge Management (PSM) facilities. Patients visiting primary HDS will be registered under ABDM through HMIS and generate an ABHA number for online medical records and patient pathway tracking. All the Healthcare facilities and providers will be registered under ABDM by generating a Health care facility ID and a Health professional ID, respectively.
  Medical officers will assess patients for the complexity of their medical needs. Patients are tagged with an ABHA number. In case a patient needs to be referred to a higher centre, the tagged patient will exit from the PHC, enter the Secondary and tertiary care grid, and can be tracked through different levels of HDS. The ABDM will help healthcare providers track the patient's moment at each level of care.
B Meso level of integration
  The distribution of patients within the secondary and tertiary HDS will be managed through the CGCR. The CGCR will act on the principles of Identification and Routing of referrals initiated through primary HDS. The CGCR acts as the hub of the Spoke Model, where the CGCR will act as a control centre for the distribution and assigning of patients referred from the Primary Gateway to integrated Secondary Capsules (SdC). Each SdC will be a replica of the other, having identical health resources (Fig. 2). Within each spoke the allocation of patients to each capsule will be done per the bucket overflow model (Fig. 3). The patients referred from PHC will be diverted to each capsule till the beds available at each SdC are filled. As soon as one capsule gets filled by patients, new patients will be allocated to the next capsule, and so on. Patients will only be referred to tertiary care through CGCR when all the SdC are occupied or if the specific patient requires a specialised tertiary level of care.
  If sick patients land in a Primary Gateway, CGCR will directly refer patients to the tertiary care system, bypassing the secondary care grid (Figs. 2 and 4).
C Micro level of integration
  Each SdC constitutes five major components, i.e., Out Patient Department (OPD), Inpatient Patient Department, Emergency, Minor Operation Theater, and ancillary and auxiliary services (Fig. 5).
  Secondary care HDS will act as a self-contained and self-equipped care centre facility available at the SdC per IPHS standards [53]. Functioning of the OPDs will be based on a proposed Triple-layered Concentric Circle Out Patient Department (TLCCO) design, planned to have *First*, ancillary and auxiliary services in the outer ring, *Second*, screening OPD in the middle ring, and *Third*, Referral and Cross Referral cases in the third innermost ring (Fig. 6). TLCCO will decrease the need to travel from one level of care to another for general and specialist outpatient care centres and will be provided under a single roof. It is envisaged to have a unidirectional design that prevents the crisscrossing of patients.
  Three-Door OPD Concept (TDO): Out-patient consultation chambers will be planned with three doors: one for entry, the second for exit, and the third for speciality or cross-consultation. Patients requiring specialist consultation will be directly entered into the inner rings through the third door. TDO will prevent the intermixing of patients and provide a unidirectional flow of patients (Fig. 6).

## Discussion

The proposed IMIH provides a framework to eliminate HDS fragmentation by providing a controlled and coordinated patient movement between the levels of care using a patient-centric approach. Our model has some key features which ensures its utility and acceptability. *First,* the patient’s movement is based on routing (referral system) and flow of data within digital platforms which are already in place. *Second*, the only entry point into the IMIH is primary care gateway, where decision is made by the ‘gatekeeper’ doctor to either keep the patient at primary HDS or refer him/her to a higher center. *Third,* the patient’s physical movement is paralleled by referrals and movement of data between primary, secondary and tertiary HDS. *Fourth,* the secondary care HDS are physically designed to enable optimal integration and continuity of care right from its entry to exit. Lastly, The IMIH also focuses on patient’s education and preventive measures during the follow-up at its nearest peripheral HDS using updated online health records, thus limiting the excessive load on tertiary care institutions.

Our review depicted that the existing IC models adopted by the developed countries were customised for their countries with limitations of having prolonged delivery time, focussed only on chronic diseases, or were economically expensive for LMICs. Also, most of the IC models were unsuitable for Indian Healthcare system as they require massive revamping of the healthcare system. Resource constraints call for planning a patient-centric IC model that can fit in the existing HDS and utilise the existing resources and infrastructure. The proposed IMIH is based on the pillars of the existing HDS developed over the years and can complement the system. The IMIH envisages improved continuity of care, better access to specialists and management timeliness, care through existing government schemes and packages by coordinated services, and improved patient flow. From a service provider’s perspective, better communication minimises work duplication, reduces staff time, and enhanced accessibility to information, which helps offer standardised care.

The IMIH introduces the Gatekeeper physicians as a primary contact point to enter the referral system. A study from California conducted to determine patients’ perception of the role of primary care physicians as first-contact care providers and coordinators of referrals and whether primary care physicians impede access to specialists depicted that almost all patients valued the role of a primary care physician as a source of first-contact care and coordinator of referrals and 75% to 91% of patients preferred to seek care initially from their primary care physicians rather than specialists [54]. Another study from Egypt narrates the problem of overuse of hospital outpatient clinics due to the lack of an effective gatekeeper system, where the price of direct hospital outpatient visits was increased, thus indirectly encouraging to visit primary care through the gatekeeper system, which resulted in a 63% decrease in direct hospital outpatient visits [55]. Although the existing healthcare delivery system in India is based on the level of care framework, the role of a Primary care physician is not based on a gatekeeper model to coordinate the referral. Once the process is in place, IMIH will help uplift the status of primary health care physicians and their role in disease management over the time, but it should be supported by adequate capacity building and standardised protocols, and continuous medical education.

The IMIH introduces a CGCR to address patient surge management centres for load-balancing and diverting patients in a coordinated manner through a gateway between 3 tiers of HDS. The CGCR will create Patient Surge Management (PSM) facilities using principles applied during the pandemic in which a three-tier arrangement of dedicated COVID-19 health facilities, including COVID Care Center (CCC); Dedicated COVID Health Centre (DCHC) and Dedicated COVID Hospital (DCH)] has been implemented in the country [56]. A similar approach was used during the COVID-19 pandemic for oxygen allocation using the oxygen demand aggregation system, Oxygen Digital Tracking System, and OxyCare app that helped coordinate demand and supply [56, 57]. The CGCR will keep a two-way track of patients referred to higher or lower levels of care and guide the patient by specifying the bed availability to the nearest referral centre. The government of West Bengal- an Eastern state of India- has started working on a particular control room at the Swasthya Bhawan to check whether the referral cases made from one medical establishment to another were required [58]. It is stated that smoothing the flow of patients in and out of institutions could help to reduce wide fluctuations in occupancy rates and prevent surges in patient visits that lead to overcrowding, poor handoffs, and delays in care [59]. CGCR is crucial in eliminating fragmentation from the HDS and providing cost-effective, acceptable, lean, and safe healthcare. The CGCR will act on the principles of Identification and Routing of referrals initiated through primary HDS. In addition, the CGCR helps share the electronic medical records with the referral facility even before the patient arrives and will help in planning necessary management. The ABDM already implemented through the National Health Authority (NHA) Government of India, provides a digital framework based on the National Digital Health Ecosystem (NDHE), which supports a continuum of care and adheres to Fast Healthcare Interoperability Resources (FHIR) guidelines of Health Level Seven International (HL7) is already in place and ready for use [60].

We further propose strengthening the secondary level of care to reduce overwhelmed tertiary care centres. In India and other LMICs, the patient load at tertiary care facilities is constantly increasing [61]. IPHS 2022 guidelines narrated the detailed plans for adequate resources in terms of infrastructure [53]. The IMIH will help properly utilise the secondary level through Load-balancing by diverting patients in a coordinated manner through CGCR primarily to secondary care capsules using the bucket overflow model, which allows the optimum utilisation of health resources and reducing pressure at the tertiary care. A study in the USA estimated that 75% to 85% of the general population only needs primary care services; 10% to 12% need referrals to short-term secondary care services. 5% to 10% of patients require specialised tertiary care services [62]. In a resource-constrained country like India, utilising available facilities to their maximum is crucial, as envisaged using the bucket overflow model, a metaphorical term used to describe a condition when all the beds are occupied in SdC, the patients are referred to the next capsule in a controlled manner. Although examples of re-routing are not directly available in healthcare, the principle of re-routing can be found in Vehicular traffic rerouting for better traffic mobility [63]. Then, the Three-Door OPD Concept (TDO) in OPDs at the SdC will provide a one-stop comprehensive patient-centric care in which patients will get all the out-patient care under a single roof without moving from one level to another. Even at a lower level of care, systematic, comprehensive care has been shown to improve outcomes for chronic diseases [64–66]. However, the better care offered at the secondary and tertiary care level lures patients to practice self-referral, leading to fragmentation of care at the level of general and speciality practitioners. This can, however, be resolved if we enforce the primary care gateway as the only entry point into the IMIH, as previous studies also show that more visits to hospitals and fewer visits to primary care facilities further lead to increased hospitalisations [11].

There are specific policy implications of this model. We visualise reduced OOPE through this model mainly by promoting better utilisation of government healthcare centres. While being patient-centric, direct and indirect, OOPE can still be reduced, *firstly*, by reducing the duplication of investigations and better utilisation of government facilities and *secondarily*, by a coordinated healthcare delivery system by ensuring continuity of care and promotion of targeted health education, self-care, and health promotion programs. By engaging the IC model in the existing healthcare delivery system, the goal of increasing utilisation of the public health sector can be achieved without massively disturbing the current framework [67]. Lastly, the model uses a lean healthcare delivery system. Lean healthcare is a management tool that minimises waste in healthcare delivery services by identifying and removing unnecessary steps that waste crucial patient serving time [68]. However, a few other critical components essential to building a successful IC model must be considered for any model to succeed. Commitment starting with solid leadership, updated IT support, and a unified budget to cover joint working between various integrated divisions is the main bottleneck that can restrain the integration. Despite a 3-tier HDS, IC in India is relatively new and has not been much developed and tested. IMIH is mostly in line with the biomedical diagnosis-centered model of care and a step towards a future biopsychosocial patient(person)-centred integration model.

### Strengths and limitations

The study has a few strengths and limitations. The IMIH focuses on a life course approach for patients, unlike other models that focus on one or the different life stages or medical conditions [39, 47, 51, 69]. Using the existing healthcare infrastructure and digital ecosystem to frame the IMIH remains the core strength. A patient-centric approach will help us to track them and ensure a continuum of care. Comparison with the established models can be seen as another strength of the study. However, as the study's primary aim was to conceptualise and develop a customised IC model for India, we have preferred a narrative review over a systematic review to emphasise more on the model than reviewing the existing models. However, narrative reviews have their own limitations regarding robustness, which should be considered while interpreting the review part of the manuscript. Lastly, we focussed only on improving the referral system to ensure the continuum of care. However, it is only one of many strategies to improve health indicators, and it is pertinent to mention that preventive and promotive interventions are the mainstay approaches that will ultimately bring lasting changes, and their role cannot be negated.

### Future research

It would be too ambitious to say that IMIH will considerably reduce the burden of chronic disease or solve the problem of resource scarcity in the HDS, which needs the active participation of the state and central governments and the adaptation of the IMIH model by the government still require elaborate discussions. Future research should thus focus on generating more evidence to support our stance. We recommend a detailed systematic review of the existing models from LMICs to generate robust evidence. Field trials and stakeholder analysis can be initiated using IMIH or similar models based on local context and ascertain the barriers and facilitators in implementing such models. At the same time, it is equally important to work on the capacity building of the manpower and establish the standard of care, ultimately defining the quality of care as envisaged through a continuum of care. The digital healthcare ecosystem is still in the infancy stages in most resource-constrained settings and should be developed further to reap the maximum benefits of the evolving technology.

## Conclusions

To conclude, the IMIH offers unique features that considers the resource constraints and local context of the LMICs while being economically viable. This model envisages a step toward UHC through the optimum utilisation of resources, ensuring a continuum of care, decreased time to care, and minimal OOPE. Coordinated and Controlled referrals augmented by transferring patient health records through digital health platforms, with standardised treatment protocols across different levels, can help improve the perceived knowledge among the healthcare staff, standardised practices, and adherence to guidelines. However, health being a state subject in India, various socio-political and legal/administrative issues warrant further discussion before implementing the IMIH.

## Data Availability

The details of the reviewed IC model are available from the corresponding author through email.

## References

[1] World Health Organization. The Global Health Observatory - Indicators. Available from: https://www.who.int/data/gho/data/indicators. Cited 2023 Oct 25.

[2] Akhtar MN, Haleem A, Javaid M (2023). Scope of health care system in rural areas under Medical 4.0 environment. Intell Pharm.

[3] Fullman N, Yearwood J, Abay SM, Abbafati C, Abd-Allah F, Abdela J (2018). Measuring performance on the Healthcare Access and Quality Index for 195 countries and territories and selected subnational locations: a systematic analysis from the Global Burden of Disease Study 2016. The Lancet..

[4] Bhore committee,1946 | National Health Portal Of India. Available from: https://www.nhp.gov.in/bhore-committee-1946_pg. Cited 2022 Aug 2.

[5] Ministry of Health and Family Welfare. Rural Health Statistics 2021 - 2022. In: Government of India. 2022. [cited 6 Nov 2023]. Available: https://hmis.mohfw.gov.in/downloadfile?filepath=publications/Rural-Health-Statistics/RHS2021-22.pdf.

[6] Ugargol AP, Mukherji A, Tiwari R (2023). In search of a fix to the primary health care chasm in India: can institutionalizing a public health cadre and inducting family physicians be the answer?. Lancet Reg Health Southeast Asia..

[7] Statistics Division (2021). Rural Health Statistics, 2020-21, Government of India Ministry of Health and Family Welfare Statistics Division.

[8] Abdi WO, Salgedo WB, Nebeb GT. Magnitude and Determinants of Self-Referral of Patients at a General Hospital, Western Ethiopia. http://www.sciencepublishinggroup.com. 2015;4:86. Available from: http://www.sciencepublishinggroup.com/j/sjcm. Cited 2023 Jan 14.

[9] Besancenot D, Sirven N, Vranceanu R (2018). A model of hospital congestion in developing countries.

[10] Kern LM, Safford MM, Slavin MJ, Makovkina E, Fudl A, Carrillo JE (2019). Patients’ and Providers’ Views on Causes and Consequences of Healthcare Fragmentation in the Ambulatory Setting: a Qualitative Study. J Gen Intern Med..

[11] Agha L, Frandsen B, Rebitzer JB. Nber working paper series causes and consequences of fragmented care delivery: theory, evidence, and public policy. 2017. Available from: http://www.nber.org/papers/w23078. Cited 2022 Aug 27.

[12] Services H, Programme D (2013). ROADMAP Strengthening people-centred health systems in the WHO European Region A Framework for Action towards Coordinated/Integrated Health Services Delivery (CIHSD).

[13] Shaw S, Rosen R, Rumbold B. What is integrated care? Research report. 2011. Available from: www.nuffieldtrust.org.uk/integratedcare. Cited 2021 Nov 8.

[14] Goodwin N (2016). Understanding Integrated Care. Int J Integr Care..

[15] Lewis RQ, Rosen R, Goodwin N, Ifer Dixon J. Where next for integrated care organisations in the English NHS?. The Nuffield Trust. Available from: https://www.nuffieldtrust.org.uk/sites/default/files/2017-01/where-next-integrated-care-english-nhs-web-final.pdf.

[16] Ham C, Murray R. Implementing the NHS five year forward view: aligning policies with the plan. 2014. Available from: https://www.kingsfund.org.uk/sites/default/files/field/field_publication_file/implementing-the-nhs-five-year-forward-view-kingsfund-feb15.pdf. Cited 2022 Jul 5.

[17] Long T, Khan AM, Chana N (2015). Achieving better value: primary care must lead on population health. Postgrad Med J..

[18] The forward view into action: New Care Models: update and initial support. 2015. Available from: https://www.england.nhs.uk/wp-content/uploads/2015/07/ncm-support-package.pdf. Cited 2022 Jul 6.

[19] WHO global strategy on people-centred and integrated health services Interim Report. 2015. Available from: https://apps.who.int/iris/bitstream/handle/10665/155002/WHO_HIS_SDS_2015.6_eng.pdf. Cited 2022 May 16.

[20] Leutz WN (1999). Five Laws for Integrating Medical and Social Services: Lessons from the United States and the United Kingdom. Milbank Q.

[21] Amelung V, Stein V, Goodwin N, Balicer R, Nolte E, Suter E. Handbook Integrated Care. Amelung V, Stein V, Goodwin N, Balicer R, Nolte E, Suter E, editors. Handbook Integrated Care. Cham: Springer International Publishing; 2017. Available from: http://link.springer.com/10.1007/978-3-319-56103-5. Cited 2021 Nov 6.

[22] Hughes G, Shaw SE, Greenhalgh T. Rethinking Integrated Care: A Systematic Hermeneutic Review of the Literature on Integrated Care Strategies and Concepts. Milbank Q . 2020; 98:446–92. Available from: https://onlinelibrary.wiley.com/doi/full/10.1111/1468-0009.12459. Cited 2022 Aug 25. 10.1111/1468-0009.12459 PMC7296432 32436330

[23] Moher D (2009). Preferred Reporting Items for Systematic Reviews and Meta-Analyses: The PRISMA Statement. Ann Intern Med..

[24] Curry N, Ham C. Clinical and service integration: the route to improved outcomes, The King’s Fund, 2010. Available from: www.kingsfund.org.uk/publications. Cited 2022 Feb 6.

[25] World Health Organization - Europe. World Health Organization -Integrated care models: an overview Working document. 2016. Available from: http://www.euro.who.int/pubrequest. Cited 2022 Feb 5.

[26] Rittenhouse DR, Shortell SM, Fisher ES (2009). Primary care and accountable care–two essential elements of delivery-system reform. N Engl J Med..

[27] Personal Health Budgets - Quarter 3 2019-20 publication - NDRS. Available from: https://digital.nhs.uk/data-and-information/publications/statistical/personal-health-budgets/2019-20-q3/personal-health-budgets-q3-2019-20#resources. Cited 2023 Mar 27.

[28] Reimers TM, Brown KM, Van Horn L, Stevens V, Obarzanek E, Hartmuller VW (1998). Maternal acceptability of a dietary intervention designed to lower children’s intake of saturated fat and cholesterol: the Dietary Intervention Study in Children (DISC). J Am Diet Assoc..

[29] DiPiero A, Dorr DA, Kelso C, Bowen JL (2008). Integrating Systematic Chronic Care for Diabetes into an Academic General Internal Medicine Resident-Faculty Practice. J Gen Intern Med..

[30] Macadam M. PRISMA: Program of Research to Integrate the Services for the Maintenance of Autonomy. A system-level integration model in Quebec. Int J Integr Care. 2015;15. Available from: /pmc/articles/PMC4583074/. cited 2022 Aug 13 10.5334/ijic.2246 PMC4583074 26417212

[31] Feachem RGA, Sekhri NK, White KL (2002). Getting more for their dollar: a comparison of the NHS with California’s Kaiser Permanente. BMJ.

[32] American Case Management Association. Definition of case management. Available from: https://www.acmaweb.org/section.aspx?sID=4. Cited 2022 Feb 6.

[33] Royal Pharmaceutical Society England. Improving care for people with long term conditions. 2016. Available from: https://www.rpharms.com/Portals/0/RPS%20document%20library/Open%20access/Policy/LTC%20-%20England.pdf. Cited 2023 Nov, 23.

[34] Gadsby EW, Segar J, Allen P, Checkland K, Coleman A, McDermott I, et al. Personal Budgets, Choice and Health: A Review of International Evidence from 11 OECD Countries. https://services.igi-global.com/resolvedoi/resolve.aspx?doi=104018/ijpphme2013070102. 1AD ;3:15–28. Available from: https://www.igi-global.com/article/personal-budgets-choice-and-health/114243. Cited 2022 Aug 20.

[35] 5 Key Functions of the Medical Home | Agency for Healthcare Research and Quality. Available from: https://www.ahrq.gov/ncepcr/tools/pcmh/implement/key-functions.html#patientCenteredHeader. Cited 2022 Feb 7.

[36] Schram AP (2010). Medical Home and the Nurse Practitioner: A Policy Analysis. J Nurs Pract.

[37] Alakeson V, Boardman J, Boland B, Crimlisk H, Harrison C, Iliffe S (2016). Debating personal health budgets. BJPsych Bull..

[38] Personal Health Budgets - Quarter 3 2019-20 publication - NHS Digital. Available from: https://digital.nhs.uk/data-and-information/publications/statistical/personal-health-budgets/2019-20-q3/personal-health-budgets-q3-2019-20#resources. Cited 2022 Feb 16.

[39] Davy C, Bleasel J, Liu H, Tchan M, Ponniah S, Brown A. Effectiveness of chronic care models: opportunities for improving healthcare practice and health outcomes: a systematic review. BMC Health Serv Res. 2015 [cited 2022 Aug 9];15. Available from: /pmc/articles/PMC4448852/10.1186/s12913-015-0854-8 PMC4448852 25958128

[40] Ham C, Glasby J, Parker H, Smith J. Altogether now? Policy options for integrating care Executive Summary Different models of integration. Health Services Management Centre. Available from: www.hsmc.bham.ac.uk. Cited 2022 Aug 11.

[41] Hébert R, Raîche M, Dubois MF, Gueye NR, Dubuc N, Tousignant M (2010). Impact of PRISMA, a coordination-type integrated service delivery system for frail older people in Quebec (Canada): A quasi-experimental study. J Gerontol B Psychol Sci Soc Sci..

[42] Åhgren B. Chain of care development in Sweden: results of a national study. Int J Integr Care. 2003 ;3. Available from: http://www.ijic.org/article/10.5334/ijic.88. Cited 2022 Aug 20. PMC1483939 16896423

[43] World Health Organization. Regional Office for Europe, European Observatory on Health Systems and Policies, Nolte, Ellen, Knai, Cécile & McKee, Martin. Managing chronic conditions: experience in eight countries. World Health Organization. Regional Office for Europe. ‎2008. https://iris.who.int/handle/10665/107920.

[44] Ahgren B, Pol Sc M, Stromberg B&. Chain of care development in Sweden: results of a national study. Int J Integr Care. 2003. 3. Available from: /pmc/articles/PMC1483939/. Cited 2022 Aug 13. PMC1483939 16896423

[45] Baker CD (2000). Cardiology: the development of a managed clinical network. BMJ..

[46] Clinical networks | National Services Scotland. Available from: https://www.nss.nhs.scot/specialist-healthcare/national-networks/what-are-national-networks/clinical-networks/. Cited 2022 Aug 14.

[47] Knai C. The editors Ellen Nolte, Hub Cordinator, European Observatory on Health Systems and Policies. Observatory Studies Series Country reports Assessing chronic disease management in European health systems. 29035490

[48] Racheli Magnezi MPKRMZMK-LMRM. Disease Management Programs: Barriers and Benefits. Am J Manag Care. 2013 ;19. Available from: https://www.ajmc.com/view/disease-management-programs-barriers-and-benefits. Cited 2022 Aug 15. 23725452

[49] Pines J, Selevan J, George M, Mcclellan M. Kaiser Permanente – California: A Model for Integrated Care for the Ill and Injured. Richard Merkin Initiative on Payment Reform and Clinical Leadership Case Study: Emergency Medicine. Available from: https://www.brookings.edu/wp-content/uploads/2016/07/KaiserFormatted_150504RH-with-image.pdf. Cited 2022 Aug 15.

[50] Jonathan B. Perlin MPMMKMHRM. The Veterans Health Administration: Quality, Value, Accountability, and Information as Transforming Strategies for Patient-Centered Care. Am J Manag Care. 2004 ;10. Available from: https://www.ajmc.com/view/nov04-1955p828-836. Cited 2022 Aug 15. 15609736

[51] Urtaran-Laresgoiti M, Álvarez-Rosete A, Nuño-Solinís R. A system-wide transformation towards integrated care in the Basque Country: A realist evaluation:. 2018 ;21:98–108. Available from: https://journals.sagepub.com/doi/10.1177/2053434518800884. Cited 2022 Aug 16.

[52] Rogers HL, Pablo Hernando S, Núñez Fernández S, Sanchez A, Martos C, Moreno M (2021). Barriers and facilitators in the implementation of an evidence-based health promotion intervention in a primary care setting: a qualitative study. J Health Organ Manag..

[53] IPHS 2022 and its layouts | National Health Systems Resource Centre. [cited 2023 Mar 5]. Available from: https://nhsrcindia.org/IPHS2022.

[54] Grumbach K, Selby JV, Damberg C, Bindman AB, Quesenberry JC, Truman A (1999). Resolving the Gatekeeper Conundrum. JAMA..

[55] Ward TR (2010). Implementing a gatekeeper system to strengthen primary care in Egypt: pilot study. East Mediterr Health J..

[56] Ministry of Health and Family Welfare. Upgradation of Healthcare System for Effective Management of COVID-19. 2021. Available from: https://pib.gov.in/PressReleasePage.aspx?PRID=1739460. Cited 2023 Jan 12.

[57] Mirza M, Verma M, Sahoo S, Roy S, Kakkar R, Singh D (2023). India’s Multi-Sectoral Response to Oxygen Surge Demand during COVID-19 Pandemic: A Scoping Review. Indian J Community Med..

[58] Special Control Room to check referral of cases by Govt hospitals. Available from: https://medicaldialogues.in/special-control-room-to-check-referral-of-cases-by-govt-hospitals. Cited 2023 Mar 8.

[59] Achieving Hospital-wide Patient Flow | IHI - Institute for Healthcare Improvement. Available from: https://www.ihi.org/resources/Pages/IHIWhitePapers/Achieving-Hospital-wide-Patient-Flow.aspx. Cited 2023 Mar 8.

[60] Home - FHIR Implementation Guide for ABDM v4.0.0. Available from: https://nrces.in/ndhm/fhir/r4/index.html. Cited 2023 Nov 21.

[61] Chavan Y, Pande B (2019). General outpatient department in tertiary care institute: A model to be adopted by medical colleges. J Family Med Prim Care..

[62] Starfield B (1994). Is primary care essential?. Lancet..

[63] de Souza AM, Braun T, Botega LC, Cabral R, Garcia IC, Villas LA (2019). Better safe than sorry: a vehicular traffic re-routing based on traffic conditions and public safety issues. J Int Serv Appl.

[64] Shojania KG, Ranji SR, McDonald KM, Grimshaw JM, Sundaram V, Rushakoff RJ (2006). Effects of quality improvement strategies for type 2 diabetes on glycemic control: a meta-regression analysis. JAMA..

[65] Gilbody S, Bower P, Fletcher J, Richards D, Sutton AJ (2006). Collaborative care for depression: a cumulative meta-analysis and review of longer-term outcomes. Arch Intern Med..

[66] Walsh JME, McDonald KM, Shojania KG, Sundaram V, Nayak S, Lewis R (2006). Quality improvement strategies for hypertension management: a systematic review. Med Care..

[67] Electronic Patient Record - an overview | ScienceDirect Topics. Available from: https://www.sciencedirect.com/topics/medicine-and-dentistry/electronic-patient-record. Cited 2022 Aug 24.

[68] What Is Lean Healthcare?. Available from: https://catalyst.nejm.org/doi/full/10.1056/CAT.18.0193. Cited 2022 Aug 25.

[69] Managing chronic conditions: experience in eight countries. Available from: https://apps.who.int/iris/handle/10665/107920. Cited 2022 Aug 15.

